# Prevalence of *PALB2 *mutations in Australasian multiple-case breast cancer families

**DOI:** 10.1186/bcr3392

**Published:** 2013-02-28

**Authors:** Zhi L Teo, Daniel J Park, Elena Provenzano, Catherine A Chatfield, Fabrice A Odefrey, Tu Nguyen-Dumont, kConFab, James G Dowty, John L Hopper, Ingrid Winship, David E Goldgar, Melissa C Southey

**Affiliations:** 1 Genetic Epidemiology Laboratory, The University of Melbourne, Grattan Street, Melbourne, Victoria 3010, Australia; 2 Department of Pathology, The University of Melbourne, Grattan Street, Melbourne, Victoria 3010, Australia; 3 Cancer Research Division, The Peter MacCallum Cancer Centre, St Andrews Place, East Melbourne, Victoria 3002, Australia; 4 Centre for Molecular, Environmental, Genetic and Analytic Epidemiology, The University of Melbourne, Level 3, 207 Bouverie Street, Carlton, VIC 3053, Australia; 5 Department of Medicine, The University of Melbourne, Grattan Street, Melbourne, Victoria 3010, and Royal Melbourne Hospital, Grattan Street, Parkville, Victoria 3050, Australia; 6 Department of Dermatology and Huntsman Cancer Institute, University of Utah School of Medicine, 30 North 1900 East, Salt Lake City, UT 84112, USA

## Abstract

**Introduction:**

Population-based studies of breast cancer have estimated that some *PALB2 *mutations confer a breast cancer risk (penetrance) comparable to the average pathogenic mutation in *BRCA2*. As this risk is of clinical relevance, we sought to identify mono-allelic *PALB2 *mutations and determine their frequencies in multiple-case breast cancer families attending Familial Cancer Clinics in Australia and New Zealand.

**Methods:**

The youngest affected woman, not known to carry a mutation in *BRCA1 *or *BRCA2*, from 747 multiple-case breast cancer families participating in kConFab were selected for *PALB2 *mutation screening. The coding and flanking intronic regions of *PALB2 *in DNA extracted from blood were screened using high-resolution melt curve analysis with Sanger sequencing confirmation. Where possible, relatives of women found to carry *PALB2 *mutations were genotyped for the family-specific mutation, mutant transcripts were characterised and breast tumours arising in mutation carriers were recalled and reviewed. Missense mutations were assessed for potential to disrupt protein function via SIFT, Align GVGD and Polyphen-2.

**Results:**

The mutation screen identified two nonsense mutations (*PALB2 *c.3113G>A in eight women and *PALB2 *c.196C>T in one woman), two frameshift mutations (*PALB2 *c.1947_1948insA and *PALB2 *c.2982_2983insT each in one woman), 10 missense variants, eight synonymous variants and four variants in intronic regions. Of the four *PALB2 *mutations identified that were predicted to produce truncated protein products, only *PALB2 *c.1947_1948insA had not previously been reported. *PALB2 *c.3113G>A and *PALB2 *c.196C>T were previously identified in the Australian population whereas *PALB2 *c.2982_2983insT was previously reported in the UK population. Transcripts derived from three of these mutant *PALB2 *alleles were vulnerable to nonsense-mediated decay. One missense mutation (*PALB2 *c.2993G>A) was predicted to disrupt protein function via the three *in silico *assessment methods applied. The majority of breast cancers arising in carriers that were available for review were high-grade invasive ductal carcinomas. **Conclusions**: About 1.5% (95% CI 0.6to 2.4) of Australasian multiple-case breast cancer families attending clinics are segregating protein-truncating mutations in *PALB2*, most being *PALB2 *c.3113G>A, p.Trp1038*. Given the prevalence, breast cancer risk, and tumour grade associated with this mutation, consideration of clinical *PALB2 *testing is warranted.

## Introduction

Genetic testing for mutations in breast cancer susceptibility genes offers some women and their families the opportunity for risk-reducing intervention, medical risk reduction and gene-targeted therapeutics [1,2]. Testing in Australia and New Zealand is usually limited to *BRCA1 *(MIM#113705), *BRCA2 *(MIM#600185) mutations and possibly those of *STK11 *(MIM#602216)*, PTEN *(MIM#601728) and *TP53 *(MIM#191170) if relevant clinical syndromic indications are observed. However, due to the rarity of these mutations in known breast cancer susceptibility genes and the fact that they account for less than 30% of the familial breast cancer risk [3], the majority of individuals at high risk of breast cancer do not carry these mutations and the families are clinically managed solely on the assessment of their cancer family history [4].

The search for additional breast cancer susceptibility genes has been of great interest and has successfully led to the identification and characterisation of *ATM *(MIM#607585) [5], *BRIP1 *(MIM#605882) [6], *CHEK2 *(MIM#604373) [7], and *PALB2 *(MIM#610355) [8]. Mutations in these genes are rare and early reports suggested that, on average, they are associated with moderate risks of breast cancer (lower than the high breast cancer risks associated with *BRCA1 *and *BRCA2 *mutations) [5,6,8,9]. However, large population-based studies of breast cancer have demonstrated that at least some mutations in these genes are associated with breast cancer risks that are comparable to the average risk associated with *BRCA2 *mutations [5,9-13].

PALB2, partner and localiser of BRCA2, is a BRCA1- and BRCA2-interacting protein critical for the homologous recombination-based repair of DNA double-strand breaks and checkpoint control functions [14-16]. Bi-allelic mutations in *PALB2 *explain an unrecognised Fanconi anemia complementation group, designated subtype N (FANCN), and have been found to convey high risk of childhood cancer [17,18]. Heterozygous germline loss-of-function mutations in *PALB2 *are associated with increased risk of breast cancer. The first *PALB2 *association study, which involved familial breast cancer cases and unaffected controls from the UK population, reported that the average estimated risk conferred by five *PALB2 *mutations is 2.3 (95% CI 1.4 to 3.9) [8] but subsequent population-based studies have estimated the risk associated with at least some *PALB2 *mutations to be much higher [12,13]. For example, *PALB2 *c.1592delT was identified in 18/1,918 (0.9%) Finnish breast cancer cases, unselected for family history, compared to 6/2,501 (0.2%) in controls (OR 3.94; 95% CI 1.5 to 12.1). Based on families of the 10 affected carriers, *PALB2 *c.1592delT was estimated to be associated with a 40% (95%CI 17 to 77) risk to age 70 [12]. Similarly, *PALB2 *c.3113G>A was identified in 5/1,403 (0.4%) unselected breast cancer cases and 0/764 (0%) unaffected controls in the Australian population. The cumulative risk estimated for *PALB2 *c.3113G>A, using the families of the five carrier cases, was 91% (95% CI 44 to 100) to age 70 [13]. These risks are comparable to the average breast cancer risk associated with carrying a *BRCA2 *mutation with penetrance to age 70 of 45% (95%CI 31 to 56) [9].

In this study, we screened for germline *PALB2 *mutations in a sample of 747 women affected with breast cancer (known not to carry a mutation in *BRCA1 *or *BRCA2*) from multiple-case breast cancer families participating in the Kathleen Cuningham Foundation Consortium for Research in Familial Breast Cancer (kConFab). This sample represents women and their families who attend Familial Cancer Clinics throughout Australia and New Zealand. We sought to ascertain the prevalence of *PALB2 *mutations in these women due to the high estimated breast cancer risk associated with at least some *PALB2 *mutations and to further consider if clinical testing for these mutations is warranted.

## Materials and methods

### *Participants*

Women and their families participating in the kConFab resource [19] were selected for this study. The youngest affected female members (designated the proband) of a multiple-case breast cancer family who had provided blood samples (for DNA preparation) and who were known not to carry a mutation in *BRCA1 *or *BRCA2 *were included. These participants would have undergone a variety of mutation testing strategies in research and diagnostic settings including Sanger sequencing through Myriad Genetics (Salt Lake City, UT, USA) [19,20]. The eligibility criteria for recruitment of families into kConFab was intended to maximise the number of living potentially high-risk individuals, including carriers of high-penetrance alleles, regardless of breast cancer status. The reports of cancer in the families were verified through medical records or by state-based cancer registries. Where possible, a copy of the final pathology report was obtained with the locations of archival and diagnostic tumour specimens for future requests of paraffin blocks and slides. A pathologist (EP) conducted pathology reviews of the available tumour material, which provided the information on tumour pathology presented in this paper. Histological grade was scored based on the Bloom-Richardson grading system modified by Elston and Ellis [21].

Data on estrogen receptor (ER), progesterone receptor (PR) and human epidermal growth factor-2 (HER2) status of the *PALB2 *mutation*-*associated tumours were collected, if available, from diagnostic laboratories and pathology reports. HER2 status was classified in accordance with clinical guidelines [22] and was considered to be positive if immunohistochemical test results were ranked 3+ (higher than normal amount of HER2 protein was present) or if HER2 gene amplification was demonstrated using fluorescence *in situ *hybridisation. An immunohistochemical test result of 1+ (normal amount of HER2 protein was present) was classified as negative for HER2 expression while an immunohistochemical test result of 2+ (moderate amount of HER2 protein was present) without a confirmatory fluorescence *in situ *hybridisation test was classified as equivocal.

All participants provided written informed consent for participation in the study. This study was approved by The University of Melbourne Human Research Ethics Committee.

### *High-resolution melt curve analysis and Sanger sequencing analysis of PALB2 coding variants*

High-resolution melt (HRM) and Sanger sequencing were performed as previously described in Southey *et al*. (2010) [13]. The coding sequences and the flanking intronic sequences of *PALB2 *[Q86YC2.1] were screened for genetic variants by HRM curve analysis and the variants were confirmed via Sanger sequencing. Genotyping of relatives of the probands found to carry *PALB2 *mutations was carried out via Sanger sequencing.

### *In silico analysis*

The *in silico *analyses of *PALB2 *variants were performed using Sorting Intolerant From Tolerant (SIFT) (J. Craig Venter Institute, Maryland, CA, USA) [23-25], Align Grantham Variation Grantham Deviation (Align GVGD) (International Agency for Research on Cancer, Lyon, France) [26-29] and Polymorphism Phenotyping version 2 (Polyphen-2) [30,31], which are freely available web-based programs. Protein multiple sequence alignment (PMSA) of PALB2 was made available through a recent publication [32] and was used in SIFT and Align GVGD.

SIFT calculates the probability that an amino acid at a position is tolerated conditional on the most frequent amino acid being tolerated by interrogating the amino acids appearing at each position in the alignment. If the normalised probability is less than the cutoff score of 0.05, the substitution is predicted to be deleterious [25].

The outputs of Align GVGD are combined to provide a seven-tiered genetic risk classifier: C0, C15, C25, C35, C45, C55, and C65 where C0 describes the category of variants least likely to be deleterious and C65 describes the category of variants most likely to be deleterious [28,29].

The HumDiv-trained data set of Polyphen-2 [31] was used for this research. The investigated mutation is categorised as probably damaging (probability score more than 0.85), possibly damaging (probability score between 0.16 and 0.85) or benign (probability score less than or equal to 0.15).

### *Reverse transcription PCR*

Epstein-Barr virus (EBV)-transformed lymphoblastoid cell lines (LCL) were cultured and prepared for RNA extraction as described by Southey *et al*. (2003) [33]. LCLs were divided into two equal portions for treatment with 100 mg/ml of cycloheximide to stabilise the transcripts for analysis [34] or no treatment. Both portions were then incubated for four hours at 37°C. Total RNA was isolated from the LCLs using the RNAqueous-4PCR kit (Ambion/Life Technologies, Carlsbad, CA, USA). Deoxyribonuclease 1 (Ambion/Life Technologies) was added to all extracted total RNA prior to their use in reverse transcription (RT) to remove any genomic DNA that could have been eluted with the RNA during the extraction process.

Primers for RT-PCR were designed using the default conditions on the Primer3 software (Whitehead Institute and Howard Hughes Medical Institute). The primers were also designed to ensure the 3' end of both the forward and reverse primers ended with a 'GC clamp' (one G or C base). To further guard against the amplification of genomic DNA, all primers except one (due to restrictions in length) were designed to span exon-exon boundaries. A *PALB2 *gene-specific primer (GSP) was used for cDNA synthesis via RT for all cell lines (Additional file 1). Primers were ordered from Geneworks (Hindmarsh, South Australia, Australia).

cDNA was synthesised via RT according to the specifications of the Thermoscript RT-PCR system kit (Life Technologies) using 800 ng of total RNA and the *PALB2 *GSP. RT was performed at 55°C for 50 mins followed by addition of RNase H and incubation for 20 mins at 37°C. Two µl of synthesised cDNA product was subsequently amplified using two units of Amplitaq Gold DNA Polymerase (Life Technologies) in the presence of 1x PCR Buffer II (Life Technologies), 1.5 mM MgCl_2 _(Life Technologies), 0.2 mM dNTP (Ambion/Life Technologies), 0.2 µM each of forward and reverse primers in a 50 µl reaction volume. PCR was performed using the PCR conditions suggested by the Thermoscript RT-PCR system kit (Life Technologies). PCR annealing temperature was chosen to be 51°C. No RT controls (extracted total RNA added in place of cDNA during RT-PCR) were added to control for contamination from genomic DNA. Platinum Supermix High Fidelity (Life Technologies) was used to limit the extent to which longer PCR products, resulting from the inclusion of intronic sequences, were not under-represented. RT-PCR was carried out in triplicate for each condition for each LCL. Multiple LCLs (derived from different study participants) carrying each of the mutations was included in the assays whenever possible and one 'non-carrier' LCL was included as an additional control.

RT-PCR products were separated using agarose gel electrophoresis on 2%, 3% or 4% agarose gels. Bands were excised from the gel and purified using the QIAquick Gel Extraction Kit (Qiagen, Hilden, Germany) according to the manufacturer's instructions prior to Sanger sequencing analysis.

The relative amounts of DNA product amplified in each RT-PCR reaction were measured by comparing the chromatogram peak heights (fluorescence signal intensities; FSI) of the variant nucleotides of the mutant alleles to the corresponding wild-type nucleotides of the wild-type alleles. After Sanger sequencing, Sequencing Analysis Software (Life Technologies) provides FSIs of each nucleotide of the target amplicon. FSIs of the variant and wild-type nucleotides at the heterozygous *PALB2 *c.196C>T position were recorded. FSIs of three wild-type nucleotides and their corresponding variant nucleotides in regions of frameshift resulting from *PALB2 *c.1947_1948insA or *PALB2 *c.2982_2983insT were recorded for each condition (cycloheximide treated or non-cycloheximide treated). FSIs of six wild-type nucleotides and their corresponding six variant nucleotides in regions of frameshift resulting from *PALB2 *c.3113G>A were recorded for each treatment condition. The same three or six nucleotides in the regions of frameshift were interrogated for each mutant transcript in the same sequencing direction. A proportion of the FSI of the variant nucleotide to that of the corresponding wild-type nucleotide was obtained. In the situations where three or six nucleotides were interrogated per treatment condition, the FSI proportions of the three or six nucleotides were averaged within a treatment condition. The FSI proportions of the technical replicates of each treatment condition were averaged. The difference in the mean FSI proportions in the cycloheximide-treated and non-cycloheximide-treated conditions indicated the semi-quantitative change in relative gene expression levels of the mutant transcripts in each treatment condition.

## Results

### *PALB2 mutation screening*

HRM screening of the *PALB2 *coding and flanking intronic regions in the selected sample of 747 DNAs identified 26 different *PALB2 *genetic variants. Of the 22 exonic variants, two were nonsense mutations resulting in predicted stop codons (*PALB2 *c.196C>T, p.Gln66*; *PALB2 *c.3113G>A, p.Trp1038*), two were frameshift mutations resulting in premature termination codons (PTC; *PALB2 *c.1947_1948insA, p.Glu650fs*13; *PALB2 *c.2982_2983insT, p.Ala995fs*16), 10 were missense variants, and the remaining eight were synonymous variants. Four variants were identified in the intronic region of *PALB2*, three of these were single-base changes and one was an insertion of three bases towards the 5' end of intron 4. 10 of the 26 variants had not been previously reported (Table 1).

The four nonsense and frameshift *PALB2 *mutations were observed in 11 (1.5%) of the 747 probands. Eight probands (0.9%) were found to carry *PALB2 *c.3113G>A while *PALB2 *c.196C>T, *PALB2 *c.1947_1948insA and *PALB2 *c.2982_2983insT were each carried by a single proband. *PALB2 *c.1947_1948insA has not been previously reported in published literature or in the National Centre for Biotechnology Information (NCBI [35]) or the e!Ensembl [36] genome databases. *PALB2 *c.2982_2983insT had not been previously reported in the Australian or New Zealand population.

### *In silico analysis*

The 10 exonic missense variants were analysed for their predicted effect on protein function using SIFT [23-25], Align GVGD [26,37], and Polyphen-2 [30,31] programs. *PALB2 *c.2993G>A, p.Gly998Glu was the only *PALB2 *variant predicted to be deleterious to protein function by all three programs (Table 2).

### *Transcript analysis*

**Figure 1:**
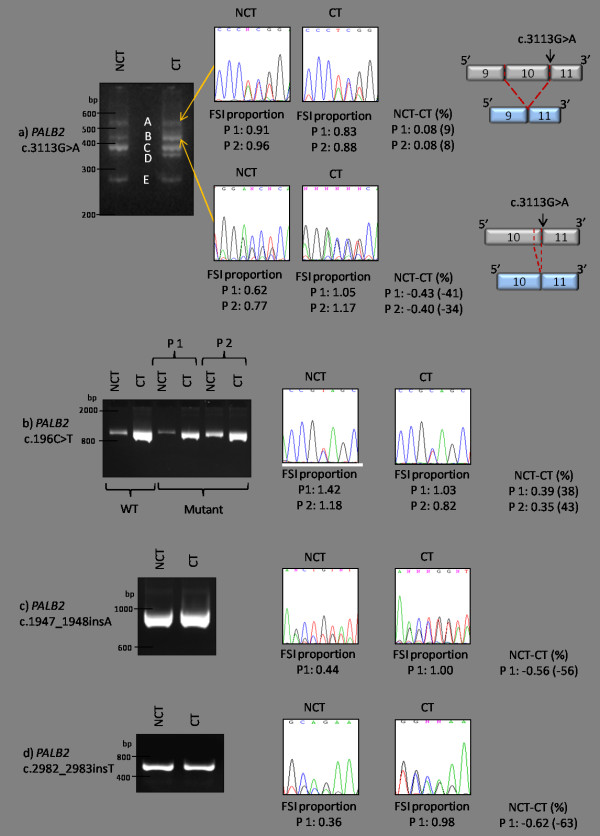
**Characterisation of *PALB2 *mutation transcripts via RT-PCR**. **(a) **Image of transcripts from *PALB2 *c.3113G>A visualised on 3% agarose gel. **(A) **Heteroduplex of the RT-PCR products of the wild-type and the *PALB2 *r.2997_3113del transcripts. **(B) **Heteroduplex of the RT-PCR products of the wild-type and *PALB2 *r.3083_3113del transcripts. **(C) **RT-PCR product of the wild-type transcript. **(D) **RT-PCR product of the *PALB2 *r.3083_3113del transcript. **(E) **RT-PCR product of the *PALB2 *r.2997_3113del transcript. FSIs of six wild-type and their corresponding six variant nucleotides at heterozygous positions were recorded for each condition (CT and NCT). Averaged proportions of FSI of the six variant nucleotides to the FSI of the six corresponding wild-type nucleotides for each condition are shown. **(b) **Image of wild-type transcripts and transcripts from *PALB2 *c.196C>T visualised on 4% agarose gel. FSIs were recorded for the variant and corresponding wild-type nucleotide at the heterozygous position. **(c) **Image of transcripts resulting from *PALB2 *c.1947_1948insA visualised on 2% agarose gel. FSIs were recorded for three variant nucleotides and their corresponding wild-type nucleotides at the heterozygous positions. **(d) **Image of transcripts from *PALB2 *c.2982_2983insT visualised on 2% agarose gel. FSIs were recorded for three variant nucleotides and their corresponding wild-type nucleotides at the heterozygous positions. NCT-CT: the difference in FSI proportions in the CT and NCT conditions indicates the semi-quantitative change in relative gene expression levels of the mutant transcripts in each condition. CT, cycloheximide treated; FSI, fluorescence signal intensity; NCT, non-cycloheximide treated; NT, nucleotide; WT, wild-type.

Figure 1 shows the outcome of the RT-PCR assays and the semi-qualitative analysis of nonsense-mediated decay (NMD) observed for the four *PALB2 *nonsense and insertion mutations identified in this study. The results of each mutation are discussed in the following sections.

### *PALB2 c.3113G>A*

*PALB2 *c.3113G>A is a nonsense mutation located at the last nucleotide of exon 10. Two alternate transcripts were observed in the RT-PCR assays. One alternate transcript involved the deletion of exon 10 (117bp*; PALB2 *r.2997_3113del, p.Gly1000_Gly1038del), and the other included a 31bp deletion in exon 10 (*PALB2 *r.3083_3113del, p.Gly1028fs*3) that resulted in a shift of reading frame and a premature termination codon (PTC) (at codon 1030). These two transcripts were also observed to travel in the agarose gel as heteroduplexes with the wild-type allele (Figure 1a, agarose gel bands A

and B). Cycloheximide treatment of LCLs carrying this mutation demonstrated that *PALB2 *r.2997_3113del does not undergo extensive NMD whereas *PALB2 *r.3083_3113del appears to be susceptible to NMD (Figure 1a).

### *PALB2 c.196C>T*

*PALB2 *c.196C>T is a nonsense mutation predicted to produce a truncated protein (p.Gln66*). Figure 1b shows that no alternate transcripts resulted from this mutation and that the cycloheximide treatment of all LCLs (both wild-type and those carrying *PALB2 *c.196C>T) had considerable impact on transcript stabilization compared to the untreated LCLs as visualised on the agarose gel. Comparison of the FSI of the mutant and wild-type alleles derived from Sanger sequencing of the RT-PCR products (semi-quantitative analysis) suggests that the wild-type transcript might be more sensitive to cycloheximide treatment than the mutant allele. Taken together, the data suggest that *PALB2 *c.196C>T is not vulnerable to NMD.

### *PALB2 c.1947_1948insA and PALB2 c.2982_2983insT*

Both *PALB2 *c.1947_1948insA and *PALB2 *c.2982**_**2983insT are frameshift mutations predicted to produce a PTC each (p.Glu650fs*13 and p.Ala995fs*16, respectively) and are not located in splice-site consensus sites. The relative levels of expression of the *PALB2 *c.1947_1948insA and *PALB2 *c.2982**_**2983insT mutant alleles in the non-cycloheximide-treated LCLs were 56% less and 63% less, respectively, than the corresponding mutant allele in the cycloheximide-treated LCLs. This suggests that transcripts arising from both these mutant alleles are subjected to NMD (Figure 1c and 1d). RT-PCR comparison of the mutant and wild-type transcripts (data not shown) indicated that neither of these *PALB2 *mutations resulted in alternate transcripts.

### *Family histories and tumour characteristics*

The relatives of women who were identified as carriers of *PALB2 *c.3113G>A, *PALB2 *c.196C>T, *PALB2 *c.1947_1948insA, and *PALB2 *c.2982_2983insT, were genotyped for the respective family mutations. Several more carriers were identified (Table 3).

*PALB2 *c.3113G>A was identified in eight probands. The median age at diagnosis (not including second primary diagnosis of breast cancer) of these probands was 48.5 years (range: 32 to 79 years). Tumour material was not available for the proband of pedigree A (Table 3a). The other seven probands were of histological grade two or three. Five of seven probands were found to have invasive ductal carcinoma as the primary histological type. One of seven probands had tubular carcinoma and the remaining proband had lobular carcinoma. The ER, PR and HER2 status of the tumours of two of these probands were available, one was ER+/PR+/HER2+ and one was ER+/PR+/HER2- (Table 3a). The families of the eight probands had at least one additional breast cancer diagnoses. Four families had six diagnoses of breast cancer. Three families were affected with several other types of cancers (Table 3a).

The proband who was found to carry *PALB2 *c.196C>T had a histological grade three, invasive ductal carcinoma diagnosed at the age of 43 years. The tumour was found to be positive for ER and PR expression but the HER2 expression status was unknown. The family had nine diagnoses of breast cancer and was also affected with six other types of cancers (Table 3b).

The proband that was identified as a carrier of *PALB2 *c.1947_1948insA had a histological grade two, pleomorphic lobular carcinoma (ER+/PR+/HER2+) diagnosed at age 42 years. There were nine additional diagnoses of breast cancers in the family and seven other types of cancers were also reported (Table 3c).

The proband that carried *PALB2 *c.2982_2983insT had a histological grade three, invasive ductal carcinoma diagnosed at age 45 years (Table 3d). Information on ER, PR and HER2 expression was not available for this individual. The family reported five breast cancers in three individuals. Other cancers in the family included sarcoma, pancreatic cancer and lymphoma.

Where possible, tumour material was also collected for some family members affected with breast cancer. In summary, *PALB2-*associated tumours are mostly high histological grade invasive ductal carcinoma (11/15; 73%). A total of 2/15 (13%) were pleomorphic lobular carcinomas, 1/15 (7%) was a lobular (classical) carcinoma and 1/15 (7%) was a tubular carcinoma.

## Discussion

High throughput screening of the *PALB2 *coding and flanking intronic regions in 747 affected women from multiple-case breast cancer families identified 26 different *PALB2 *genetic variants.

The four mutations that were predicted to produce truncated protein product were identified in a total of 11 (1.5%) of the 747 women screened. Eight of these women were found to carry *PALB2 *c.3113G>A that has previously been identified in British, Australian and American women affected with breast cancer [8,13,38,39] and is associated with an estimated 91% (95% CI 44 to 100) cumulative risk of breast cancer to age 70. We report this and the carrier frequency of PALB2 c.3113G>A in the kConFab resource in Southey *et al*., (2010) [13]. *PALB2 *c.2982_2983insT has previously been identified in one of 923 women from multiple-case breast cancer families screened for *PALB2 *mutations in a UK study (and not in 1,084 unaffected women) [8]. *PALB2 *c.196C>T has previously been reported to be carried by 2/972 (0.2%) and 1/70 (1.4%) familial breast cancer cases (and none in controls) in Australian and USA studies, respectively [38,39]. The *PALB2 *c.1947_1948insA mutation has not been previously reported.

Only one of 10 missense variants identified in this study was predicted to be deleterious by SIFT, Align GVGD and Polyphen-2. This variant, *PALB2 *c.2993G>A, has previously been identified in studies examining the role of *PALB2 *in multiple-case breast cancer families that are not known to carry *BRCA1 *or *BRCA2 *mutations. A study involving individuals in the UK population found the minor allele frequency of *PALB2 *c.2993G>A to be 33/1846 (1.8%) for familial breast cancer cases and 44/2168 (2.0%) for unaffected controls [8]. Similar carrier frequencies of this *PALB2 *variant in breast cancer cases and unaffected controls were also observed in the Finnish population [40]. The results of these studies provide no evidence to suggest that *PALB2 *c.2993G>A is associated with breast cancer risk. These finding are consistent with the report of Tischkowitz *et al*. (2012) in that we find no evidence that rare *PALB2 *missense mutations strongly influence breast cancer risk [32].

The four mutations that were predicted to produce truncated protein products were assessed for their effects on splicing and gene expression. *PALB2 *c.3113G>A was observed to lead to altered splicing of transcripts. In this study, we identified two mutant transcripts that disrupt the tenth coding exon of *PALB2*. Casadei *et al*. (2011) conducted a similar analysis and detected a third transcript (designated p.Trp1038*) in extremely low abundance (4%) [38]. The reason for the apparent discrepancy between the two studies could lie in the methodological approaches utilized as EBV transformation of LCLs, RT-PCR, gel electrophoresis and Sanger sequencing all have small inherent margins to miss transcripts that are present in low quantities. It is also possible that distinct genetic background inherent to the different LCLs (derived from different people) could contribute to the different sensitivities of transcript detection between the two studies. *PALB2 *c.196C>T, *PALB2 *c.1947_1948insA, and *PALB2 *c.2982_2983insT were not found to alter splicing.

The transcripts that contained PTCs occurring more than 55 nucleotides from the 3' exon-exon junction resulted in decreased gene expression via NMD with the exception of *PALB2 *c.196C>T. The current model for mammalian NMD postulates that NMD targets are recognised through exon junction complex (EJC) of proteins that are deposited approximately 20 to 24 nucleotides upstream of exon-exon junctions and are not removed by the ribosome after the first round of translation [41-44]. NMD-resistant PTC-containing mRNAs based on the recognition of EJCs have previously been reported for several genes [45-47]. It has since been postulated that mammalian EJCs evolved to function as NMD enhancers and that the basic EJC-independent mechanism proposed for NMD in *Saccharomyces cerevisiae*, in which the close proximity of poly(A)-binding proteins inhibit NMD [48,49], is still conserved in higher eukaryotes [50,51]. The basic EJC-independent mechanism has been observed in humans [52]. The proximity of the PTC and the poly(A) tail depends on the number of nucleotides and/or the physical distance between them. The latter depends on the three-dimensional structure of the 3' untranslated region and/or the mRNA molecule that could be affected by intramolecular base pairing, the interaction of the mRNA with RNA-binding proteins and/or the interactions between the proteins involved in translation events. In the context of this study, the three-dimensional structure of mutant *PALB2 *transcripts could result in the close proximity of the poly(A) tail and the PTC resulting from *PALB2 *c.196C>T, therefore, attenuating NMD. The presence of truncated proteins in cells from mutation carriers requires further experimental validation.

**Figure 2:**
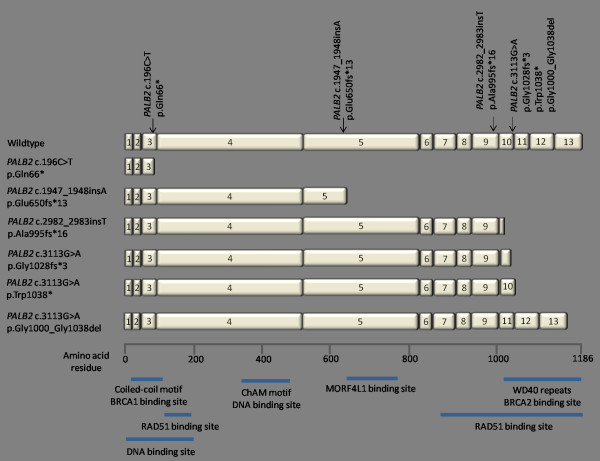
**PALB2 protein structure, binding sites of protein partners and transcript alterations due to *PALB2 *mutations**. Schematic diagram of the PALB2 protein showing its predicted functional domains, binding sites of its protein partners and the mutant transcripts resulting from *PALB2 *mutations identified in this study.

Previous studies localised amino acids 21 to 39 of BRCA2 as the region which interacted with PALB2 amino acids 1022 to 1186 [14,53,54]. The evolutionarily conserved coiled-coil domain at the N-terminus of PALB2 (amino acids 6 to 90; Figure 2) interacts with a region of BRCA1 (amino acid 1393 to 1476), which also contains a conserved coiled-coil domain [15,16]. RAD51 and MORF4L1 are also binding partners of PALB2 [55-57]. Amino acid residues in the regions of 101 to 184 and 850 to 1186 of PALB2 bind to RAD51 and, in the presence of BRCA2, guide it to chromosomal lesions and enhance its performance in initiating DNA repair [56]. MORF4L1 binds to a region included in amino acid residues 611 to 764 of PALB2 [55,57] and has been suggested to mediate DNA damage response functions of the BRCA complex in chromatin [57]. *PALB2 *c.196C>T, *PALB2 *c.1947_1948insA, *PALB2 *c.2982_2983insT and *PALB2 *c.3113G>A would affect the binding between PALB2 and at least one of its binding partners (if they encode stable prematurely truncated proteins). Subsequent potential consequences include impaired homologous recombination repair of DNA double-strand breaks [14-16,56,57]. Functional assays are required to elucidate the full extent of the disruption caused by *PALB2 *mutations to the function(s) of PALB2 and on their impact on homologous recombination repair.

The families of several probands identified in this study as carriers of frameshift or nonsense mutations of *PALB2 *were observed to include numerous diagnoses of other cancers. Leukaemia, lymphoma, melanoma, and cancers of the bowel, colon, endometrium, lung, ovary, pancreas, and prostate were repeatedly observed (three diagnoses or more) in and across families (data not shown). *PALB2 *mutations have been associated with predisposition to pancreatic [58-60] and ovarian cancers [61,62], which were both observed in families identified in our study. The possible implication of *PALB2 *as a predisposition gene in other cancers would need to be further investigated. Given the rarity of *PALB2 *mutations, studies that involve large numbers of *PALB2 *mutation carriers would be required and could be facilitated though international efforts to combine data sets.

The tumour pathology of breast cancers arising in women who carry germline nonsense or frameshift mutations in *PALB2 *was examined. Our study found that the majority of tumours that were available for analysis were high histological grade invasive ductal carcinomas (11/15; 73%), two (13%) were pleomorphic lobular carcinomas, one (7%) was a lobular (classical) carcinoma and one (7%) was a tubular-type carcinoma. Although previous studies had noted that breast cancers arising in *PALB2 *mutation carriers were more likely to be ER-/PR-/HER2- (triple negative) [61,63-65], the triple-negative receptor status was not observed in our study. Further work is warranted to examine the pathology of breast cancers arising in *PALB2 *mutation carriers.

*PALB2 *mutation detection was conducted with the application of HRM analysis involving all coding and flanking intronic regions of the gene. Methods specific for the detection of large genomic rearrangements were not applied. Prior reports demonstrate that such mutations in *PALB2 *are extremely rare and thus omission of such an analysis is unlikely to significantly impact on this study [60].

## Conclusion

We report the identification of two nonsense and two frameshift mutations of *PALB2 *in 1.5% of familial breast cancer cases recruited from Familial Cancer Clinics in Australia and New Zealand. Although rare, *PALB2 *mutations have been shown to confer high risks for the development of breast cancer [12,13,66]. Our data, together with that of many others [8,32,38,67-69] have shown that the prevalence of *PALB2 *mutations in the context of multiple-case breast cancer families is potentially relevant to their clinical management. Data support the inclusion of *PALB2 *in multi-gene panels, screened by targeted massively parallel sequencing, which are gradually being introduced as part of genetic testing. This will enable *PALB2 *mutations carriers to be provided with the best available prevention and clinical management, including screening recommendations.

## Abbreviations

Align GVGD, Align Grantham Variation Grantham Deviation; EBV, Epstein-Barr virus; EJC, exon junction complex; ER, estrogen receptor; FANCN, Fanconi anemia complementation group, designated subtype N; FSI, fluorescence signal intensities; GSP, gene-specific primer; HER2, human epidermal growth factor-2; HRM, high-resolution melt; kConFab, Kathleen Cuningham Foundation Consortium for Research in Familial Breast Cancer; LCL, lymphoblastoid cell lines; NCBI, National Centre for Biotechnology Information; NMD, nonsense-mediated decay; PMSA, protein multiple sequence alignment; Polyphen-2, polymorphism phenotyping version 2; PR, progesterone receptor; PTC, premature termination codon; RT, reverse transcription; SIFT, Sorting Intolerant From Tolerant.

## Competing interests

The authors declare that they have no competing interests.

## Authors' contributions

ZLT conducted the laboratory-based work, analysed the data, contributed to obtaining grant funding, co-wrote the manuscript and approved the final version. DJP supervised aspects of the bench work, contributed to obtaining grant funding, co-wrote the manuscript and approved the final version. EP conducted pathology review of the histopathology slides, independently graded and typed the PALB2-associated tumours, analysed the data, co-wrote the manuscript and approved the final version. TN-D contributed to data analysis and in obtaining grant funding, co-wrote the manuscript and approved the final version. CAC conducted the tissue culture aspects of this bench work and approved the final version of the manuscript. JGD contributed to obtaining all necessary approvals and clearances to conduct the research, contributed to obtaining grant funding, supervised aspects of the research, contributed to the analysis of the data, co-wrote the manuscript and approved the final version. kConFab provided the research resource utilised in this report. FAO supervised aspects of the bench work, contributed to obtaining grant funding, co-wrote the manuscript and approved the final version. JLH contributed to obtaining all necessary approvals and clearances to conduct the research, contributed to obtaining grant funding, supervised aspects of the research, contributed to the analysis of the data, co-wrote the manuscript and approved the final version. IW contributed to obtaining all necessary approvals and clearances to conduct the research, contributed to obtaining grant funding, supervised aspects of the research, contributed to the analysis of the data, co-wrote the manuscript and approved the final version. DEG contributed to obtaining all necessary approvals and clearances to conduct the research, contributed to obtaining grant funding, supervised aspects of the research, contributed to the analysis of the data, co-wrote the manuscript and approved the final version. MCS has worked to establish and maintain the research resources of kConFab, conceived and designed the study, obtained all necessary approvals and clearances to conduct the research, obtained grant funding, supervised the research, analysed the data, co-wrote the manuscript and approved the final version.

**Table 1:** *PALB2 *variants identified in female participants of the kConFab resource.

	Nucleotide change	Protein change	rs number	Frequency (*n *= 747)	%	References
Exonic variants						

Nonsense	c.196C>T	p. Gln66*	rs180177083	1	0.1	Casadei *et al*. [38]; Wong *et al*. [39]

	c.3113G>A	p.Trp1038*	rs180177132	7	0.9	Rahman *et al*. [8]; Casadei *et al*. [38]; Wong *et al*. [39]

Frameshift	c.1947_1948insA	p. Glu650fs*13	-	1	0.1	-

	c.2982_2983insT	p. Ala995fs*16	rs180177127	1	0.1	Rahman *et al*. [8]; Bogdanova *et al*.[70]

Missense	c.90G>T	p.Lys30Asn	-	1	0.1	-

	c.94C>G	p.Leu32Val	rs151316635	1	0.1	-

	c.596A>G	p.Asp219Gly	rs45594034	1	0.1	Rahman *et al*. [8]; Hellebrand *et al*. [40]; Dansonka-Mieskowska *et al*.[61]

	c.956C>A	p.Ser319Tyr	-	1	0.1	-

	c.1010T>C	p.Leu337Ser	rs45494092	25	3.3	Rahman *et al*. [8]; Hellebrand *et al*. [40];

	c.1475G>T	p.Gly492Val	-	1	0.1	-

	c.1676A>G	p.Gln559Arg	rs152451	72	9.6	Rahman *et al*. [8]; Hellebrand *et al*. [40]; Garcia *et al*. [63]; Bogdanova *et al*.[70]

	c.2014G>C	p.Glu672Gln	rs45532440	51	6.8	Rahman *et al*. [8]; Hellebrand *et al*. [40]; Garcia *et al*. [63]; Bogdanova *et al*.[70].; Dansonka-Mieskowska *et al*.[61]

	c.2590C>T	p.Pro864Ser	rs45568339	1	0.1	Rahman *et al*. [8]; Hellebrand *et al*. [40]; Garcia *et al*. [63];

	c.2993G>A	p.Gly998glu	rs45551636	17	2.3	Rahman *et al*. [8]; Hellebrand *et al*. [40]; Garcia *et al*. [63]; Bogdanova *et al*.[70]

Synonymous	c.1431C>T	p.Thr477Thr	-	1	0.1	-

	c.1470C>T	p.Pro490Pro	rs45612837	1	0.1	Rahman *et al*. [8]; Bogdanova *et al*.[70]

	c.1572A>G	p.Ser524Ser	rs45472400	4	0.5	Rahman *et al*. [8]; Hellebrand *et al*. [40]; Garcia *et al*. [63]; Bogdanova *et al*.[70]

	c.1935G>A	p.Glu645Glu	rs141707455	1	0.1	Hellebrand *et al*. [40];

	c.2469C>A	p.Leu823Leu	-	1	0.1	-

	c.2823C>A	p.Ile941Ile	-	1	0.1	-

	c.3300T>G	p.Thr1100Thr	rs45516100	45	6	Rahman *et al*. [8]; Hellebrand *et al*. [40]; Erkko *et al*. [71]; Garcia *et al*. [63]; Bogdanova *et al*.[70] Dansonka-Mieskowska *et al*.[61]

	c.3321G>A	p.Leu1107Leu	-	1	0.1	-

Intronic variants						

	c.-47G>A	-	rs8053188	17	2.3	Hellebrand *et al*. [40]; Garcia *et al*. [63];

	c.212-58A>C	-	rs80291632	37	5	Garcia *et al*. [63]; Dansonka-Mieskowska *et al*.[61]

	c.1684+41_42insTGA	-	-	2	0.3	-

	c.2834+12C>T	-	-	1	0.1	-

**Table 2:** Predicting the effects of *PALB2 *missense variants on protein function using three *in silico *methods.

Nucleotide change	Protein change	Carrier frequency (%)	SIFT ^a^	Align GVGD ^b^	Polyphen ^c^
c.90G>T	p.Lys30Asn	1 (0.1)	Affect protein function; *P *= 0.04	Class C0	Probably damaging 0.999

c.94C>G	p.Leu32Val	1 (0.1)	Tolerated; *P *= 0.37	Class C0	Probably damaging 1.000

c.596A>G	p.Asp219Gly	1 (0.1)	Tolerated; *P *= 0.65	Class C0	Benign 0.000

c.956C>A	p.Ser319Tyr	1 (0.1)	Tolerated; *P *= 0.91	Class C0	Possibly damaging 0.589

c.1010T>C	p.Leu337Ser	25 (3.3)	Tolerated; *P *= 0.59	Class C0	Benign 0.291

c.1475G>T	p.Gly492Val	1 (0.1)	Tolerated; *P *= 0.17	Class C0	Benign 0.161

c.1676A>G	p.Gln559Arg	72 (9.6)	Tolerated; *P *= 0.57	Class C0	Benign 0.000

c.2014G>C	p.Glu672Gln	51 (6.8)	Tolerated; *P *= 0.48	Class C0	Benign 0.225

c.2590C>T	p.Pro864Ser	1 (0.1)	Tolerated; *P *= 0.68	Class C0	Possibly damaging 0.578

c.2993G>A ^*^	***p.Gly998Glu***	***17 (0.9)***	***Affect protein function; p = 0.00***	***Class C65***	***Probably damaging 1.000***

^*^Predicted to affect protein function by all three programs. ^a^Kumar *et al*.(2009) [24]; Ng *et al *(2003) [25]. ^b^Tavtigian *et al*. (2006) [27]; Mathe *et al*. (2006) [28]; Tavtigian *et al*. (2008) [29]. ^c^Adzhubei *et al*. (2010) [31].

**Table 3:** Family cancer histories of probands, carriers of *PALB2 *mutations, and the histopathology features of *PALB2*-associated tumours.

Mutation	Pedigree	Breast cancer diagnoses	Grade	Histological type (1°)	ER	PR	HER2
a) *PALB2 *c.3113G>A	A	Proband **+**					
		
		P.FCOR **+**					
		
		P.cousin **-**					
		
		P.aunt					
	
	B	Proband **+**	3	IDC	pos	neg	Eqv
		
		P.aunt					
		
		P.cousin **-**					
		
		P.cousin					
		
		P.g.mother					
	
	C	Proband **+**	1	Tubular	pos	pos	neg
		
		Sister **+**	3	IDC	pos	neg	
		
		P.aunt					
		
		M.aunt					
		
		M.cousin					
	
	D	Proband **+**	2	IDC*			
		
		Sister **+**	1	IDC			
		
		P.cousin **+**					
		
		P.cousin					
		
		P.aunt					
		
		P.aunt					
	
	E	Proband **+**	3	IDC	neg	neg	Eqv
		
		Sister					
		
		M.cousin **+**					
		
		M.cousin					
		
		M.cousin **+**	2	Pl. lobular	pos	pos	
		
		M.aunt					
	
	F	Proband **+**	3	IDC	pos	pos	pos
	
	G	Proband **+**	2	Lobular			
		
		Sister					
	
	H	Proband **+**	2	IDC	pos	neg	Eqv
		
		Sister **+**	2	IDC	pos	pos	
		
		Mother **-**					
		
		M.g.mother					

b) *PALB2 *c.196C>T	I	Proband **+**	3	IDC	pos	pos	
		
		Mother **-**					
		
		P.cousin					
		
		P.cousin					
		
		P.g.-g.mother					
		
		P.g.aunt					
		
		P.g.aunt					
		
		P.g.aunt					

c) *PALB2 *c.1947_1948insA	J	Proband **+**	2	Pl. lobular	pos	pos	pos
		
		Sister					
		
		M.cousin					
		
		M.cousin					
		
		M.FCOR					
		
		M.g.Aunt					
		
		P.cousin					
		
		Sister in law					

d) *PALB2 *c.2982_2983insT	K	Proband **+**	3	IDC			
		
		Sister **+**	2-3	IDC	pos	neg	pos
		
		M.aunt					

Histopathology and carrier status information were presented where available. *Secondary histological type is lobular carcinoma. Dx, diagnosis; 1°, primary; ER, estrogen receptor; PR, progesterone receptor; HER2, human epidermal growth factor-2; P, paternal; M, maternal; G, grand; G-G, great-grand; FCOR, first cousin once removed; IDC, invasive ductal carcinoma; Pos, positive; Neg, negative; +, genotyped and identified to be a carrier of the respective *PALB2 *mutation; -, genotyped and found to not be a carrier of a *PALB2 *mutation; Pl, pleomorphic; Uk, unknown; Eqv, equivocal, tested to be 2+ by immunohistochemistry but not confirmed by fluorescence *in situ *hybridisation

## Supplementary Material

Additional file 1 **Primer sequences used for reverse transcription PCR**. Tabular data, Excel spreadsheet, .xls. Click here for file

## References

[B1] Tutt A Robson M Garber JE Domchek SM Audeh MW Weitzel JN Friedlander M Arun B Loman N Schmutzler RK Wardley A Mitchell G Earl H Wickens M Carmichael J Oral poly(ADP-ribose) polymerase inhibitor olaparib in patients with BRCA1 or BRCA2 mutations and advanced breast cancer: a proof-of-concept trial Lancet 2010 376 235 244 10.1016/S0140-6736(10)60892-6 20609467

[B2] Ashworth A A synthetic lethal therapeutic approach: poly(ADP) ribose polymerase inhibitors for the treatment of cancers deficient in DNA double-strand break repair J Clin Oncol 2008 26 3785 3790 10.1200/JCO.2008.16.0812 18591545

[B3] Mavaddat N Antoniou AC Easton DF Garcia-Closas M Genetic susceptibility to breast cancer Mol Oncol 2010 4 174 191 10.1016/j.molonc.2010.04.011 20542480 PMC5527934

[B4] National Breast and Ovarian Cancer Centre Advice about familial aspects of breast cancer and epithelial ovarian cancer: a guide for health professionals Book Advice about familial aspects of breast cancer and epithelial ovarian cancer: A guide for health professionals 2010 National Breast and Ovarian Cancer Centre

[B5] Bernstein JL Teraoka S Southey MC Jenkins MA Andrulis IL Knight JA John EM Lapinski R Wolitzer AL Whittemore AS West D Seminara D Olson ER Spurdle AB Chenevix-Trench G Giles GG Hopper JL Concannon P Population-based estimates of breast cancer risks associated with ATM gene variants c.7271T > G and c.1066-6T > G (IVS10-6T > G) from the breast cancer family registry Hum Mutat 2006 27 1122 1128 10.1002/humu.20415 16958054

[B6] Seal S Thompson D Renwick A Elliott A Kelly P Barfoot R Chagtai T Jayatilake H Ahmed M Spanova K North B McGuffog L Evans DG Eccles D; Breast Cancer Susceptibility Collaboration (UK) Easton DF Stratton MR Rahman N Truncating mutations in the Fanconi anemia J gene BRIP1 are low-penetrance breast cancer susceptibility alleles Nat Genet 2006 38 1239 1241 10.1038/ng1902 17033622

[B7] Meijers-Heijboer H van den Ouweland A Klijn J Wasielewski M de Snoo A Oldenburg R Hollestelle A Houben M Crepin E van Veghel-Plandsoen M Elstrodt F van Duijn C Bartels C Meijers C Schutte M McGuffog L Thompson D Easton D Sodha N Seal S Barfoot R Mangion J Chang-Claude J Eccles D Eeles R Evans DG Houlston R Murday V Narod S Peretz T Low-penetrance susceptibility to breast cancer due to CHEK2*1100delC in noncarriers of BRCA1 or BRCA2 mutations Nat Genet 2002 31 55 59 10.1038/ng879 11967536

[B8] Rahman N Seal S Thompson D Kelly P Renwick A Elliott A Reid S Spanova K Barfoot R Chagtai T Jayatilake H McGuffog L Hanks S Evans DG Eccles D; Breast Cancer Susceptibility Collaboration (UK) Easton DF Stratton MR PALB2, which encodes a BRCA2-interacting protein, is a breast cancer susceptibility gene Nat Genet 2007 39 165 167 10.1038/ng1959 17200668 PMC2871593

[B9] Antoniou A Pharoah PDP Narod S Risch HA Eyfjord JE Hopper JL Loman N Olsson H Johannsson O Borg A Pasini B Radice P Manoukian S Eccles DM Tang N Olah E Anton-Culver H Warner E Lubinski J Gronwald J Gorski B Tulinius H Thorlacius S Eerola H Nevanlinna H Syrjäkoski K Kallioniemi OP Thompson D Evans C Peto J Average risks of breast and ovarian cancer associated with BRCA1 or BRCA2 mutations detected in case series unselected for family history: A combined analysis of 22 studies Am J Hum Genet 2003 72 1117 1130 10.1086/375033 12677558 PMC1180265

[B10] Goldgar DE Healey S Dowty JG Da Silva L Chen X Spurdle AB Terry MB Daly MJ Buys SM Southey MC Andrulis I John EM BCFR kConFab Khanna KK Hopper JL Oefner PJ Lakhani S Chenevix-Trench G Rare variants in the ATM gene and risk of breast cancer Breast Cancer Res 2011 13 R73 10.1186/bcr2919 21787400 PMC3236337

[B11] Byrnes GB Southey MC Hopper JL Are the so-called low penetrance breast cancer genes, ATM, BRIP1, PALB2 and CHEK2, high risk for women with strong family histories? Breast Cancer Res 2008 10 208 10.1186/bcr2099 18557994 PMC2481495

[B12] Erkko H Dowty JG Nikkilä J Syrjäkoski K Mannermaa A Pylkäs K Southey MC Holli K Kallioniemi A Jukkola-Vuorinen A Kataja V Kosma VM Xia B Livingston DM Winqvist R Hopper JL Penetrance analysis of the PALB2 c.1592delT founder mutation Clin Cancer Res 2008 14 4667 4671 10.1158/1078-0432.CCR-08-0210 18628482

[B13] Southey MC Teo ZL Dowty JG Odefrey FA Park DJ Tischkowitz M Sabbaghian N Apicella C Byrnes GB Winship I Baglietto L Giles GG Goldgar DE Foulkes WD Hopper JL; kConFab for the Beast Cancer Family Registry A PALB2 mutation associated with high risk of breast cancer Breast Cancer Res 2010 12 R109 10.1186/bcr2796 21182766 PMC3046454

[B14] Xia B Sheng Q Nakanishi K Ohashi A Wu JM Christ N Liu XG Jasin M Couch FJ Livingston DM Control of BRCA2 cellular and clinical functions by a nuclear partner, PALB2 Molecular Cell 2006 22 719 729 10.1016/j.molcel.2006.05.022 16793542

[B15] Sy SMH Huen MSY Chen JJ PALB2 is an integral component of the BRCA complex required for homologous recombination repair Proc Natl Acad Sci USA 2009 106 7155 7160 10.1073/pnas.0811159106 19369211 PMC2678481

[B16] Zhang F Ma JL Wu JX Ye L Cai H Xia B Yu XC PALB2 links BRCA1 and BRCA2 in the DNA-damage response Current Biology 2009 19 524 529 10.1016/j.cub.2009.02.018 19268590 PMC2750839

[B17] Reid S Schindler D Hanenberg H Barker K Hanks S Kalb R Neveling K Kelly P Seal S Freund M Wurm M Batish SD Lach FP Yetgin S Neitzel H Ariffin H Tischkowitz M Mathew CG Auerbach AD Rahman N Biallelic mutations in PALB2 cause Fanconi anemia subtype FA-N and predispose to childhood cancer Nat Genet 2007 39 162 164 10.1038/ng1947 17200671

[B18] Xia B Dorsman JC Ameziane N de Vries Y Rooimans MA Sheng Q Pals G Errami A Gluckman E Llera J Wang W Livingston DM Joenje H de Winter JP Fanconi anemia is associated with a defect in the BRCA2 partner PALB2 Nat Genet 2007 39 159 161 10.1038/ng1942 17200672

[B19] Mann GJ Thorne H Balleine RL Butow PN Clarke CL Edkins E Evans GM Fereday S Haan E Gattas M Giles GG Goldblatt J Hopper JL Kirk J Leary JA Lindeman G Niedermayr E Phillips KA Picken S Pupo GM Saunders C Scott CL Spurdle AB Suthers G Tucker K Chenevix-Trench G; Kathleen Cuningham Consortium for Research in Familial Breast Cancer Analysis of cancer risk and BRCA1 and BRCA2 mutation prevalence in the kConFab familial breast cancer resource Breast Cancer Res 2006 8 R12 10.1186/bcr1377 16507150 PMC1413975

[B20] John EM Hopper JL Beck JC Knight JA Neuhausen SL Senie RT Ziogas A Andrulis IL Anton-Culver H Boyd N Buys SS Daly MB O'Malley FP Santella RM Southey MC Venne VL Venter DJ West DW Whittemore AS Seminara D; Breast Cancer Family Registry The Breast Cancer Family Registry: an infrastructure for cooperative multinational, interdisciplinary and translational studies of the genetic epidemiology of breast cancer Breast Cancer Res 2004 6 R375 R389 10.1186/bcr801 15217505 PMC468645

[B21] Elston CW Ellis IO Pinder SE Pathological prognostic factors in breast cancer Crit Rev in Oncol Hematol 1999 31 209 223 10.1016/S1040-8428(99)00034-7 10532196

[B22] Wolff AC Hammond ME Schwartz JN Hagerty KL Allred DC Cote RJ Dowsett M Fitzgibbons PL Hanna WM Langer A McShane LM Paik S Pegram MD Perez EA Press MF Rhodes A Sturgeon C Taube SE Tubbs R Vance GH van de Vijver M Wheeler TM Hayes DF American Society of Clinical Oncology College of American Pathologists American Society of Clinical Oncology/College of American Pathologists guideline recommendations for human epidermal growth factor receptor 2 testing in breast cancer J Clin Oncol 2007 25 118 145 17159189 10.1200/JCO.2006.09.2775

[B23] SIFT http://sift.jcvi.org/

[B24] Kumar P Henikoff S Ng PC Predicting the effects of coding non-synonymous variants on protein function using the SIFT algorithm Nat Protoc 2009 4 1073 1082 10.1038/nprot.2009.86 19561590

[B25] Ng PC Henikoff S SIFT: predicting amino acid changes that affect protein function Nucleic Acids Res 2003 31 3812 3814 10.1093/nar/gkg509 12824425 PMC168916

[B26] Align GVGD http://agvgd.iarc.fr/

[B27] Tavtigian SV Deffenbaugh AM Yin L Judkins T Scholl T Samollow PB de Silva D Zharkikh A Thomas A Comprehensive statistical study of 452 BRCA1 missense substitutions with classification of eight recurrent substitutions as neutral J Med Genet 2006 43 295 305 16014699 10.1136/jmg.2005.033878 PMC2563222

[B28] Mathe E Olivier M Kato S Ishioka C Hainaut P Tavtigian SV Computational approaches for predicting the biological effect of p53 missense mutations: a comparison of three sequence analysis based methods Nucleic Acids Res 2006 34 1317 1325 10.1093/nar/gkj518 16522644 PMC1390679

[B29] Tavtigian SV Byrnes GB Goldgar DE Thomas A Classification of rare missense substitutions, using risk surfaces, with genetic- and molecular-epidemiology applications Hum Mutat 2008 29 1342 1354 10.1002/humu.20896 18951461 PMC3938023

[B30] PolyPhen-2: prediction of functional effects of human nsSNPs http://genetics.bwh.harvard.edu/pph2/

[B31] Adzhubei IA Schmidt S Peshkin L Ramensky VE Gerasimova A Bork P Kondrashov AS Sunyaev SR A method and server for predicting damaging missense mutations Nat Methods 2010 7 248 249 10.1038/nmeth0410-248 20354512 PMC2855889

[B32] Tischkowitz M Capanu M Sabbaghian N Li L Liang X Vallée MP Tavtigian SV Concannon P Foulkes WD Bernstein L; WECARE Study Collaborative Group Bernstein JL Begg CB Rare germline mutations in PALB2 and breast cancer risk: a population-based study Hum Mutat 2012 33 674 680 10.1002/humu.22022 22241545 PMC3767757

[B33] Southey MC Tesoriero A Young MA Holloway AJ Jenkins MA Whitty J Misfud S McLachlan SA Venter DJ Armes JE A specific GFP expression assay, penetrance estimate, and histological assessment for a putative splice site mutation in BRCA1 Hum Mutat 2003 22 86 91 10.1002/humu.10224 12815598

[B34] Bateman JF Freddi S Lamande SR Byers P Nasioulas S Douglas J Otway R Kohonen-Corish M Edkins E Forrest S Reliable and sensitive detection of premature termination mutations using a protein truncation test designed to overcome problems of nonsense-mediated mRNA instability (vol 13, pg 311, 1998) Hum Mutat 1999 14 86 86 10.1002/(SICI)1098-1004(1999)13:4<311::AID-HUMU8>3.0.CO;2-P 10220145

[B35] NCBI http://www.ncbi.nlm.nih.gov/

[B36] e!Ensembl http://asia.ensembl.org/index.html

[B37] Tavtigian SV Greenblatt MS Lesueur F Byrnes GB In silico analysis of missense substitutions using sequence-alignment based methods Hum Mutat 2008 29 1327 1336 10.1002/humu.20892 18951440 PMC3431198

[B38] Casadei S Norquist BM Walsh T Stray S Mandell JB Lee MK Stamatoyannopoulos JA King MC Contribution of inherited mutations in the BRCA2-interacting protein PALB2 to familial breast cancer Cancer Res 2011 71 2222 2229 10.1158/0008-5472.CAN-10-3958 21285249 PMC3059378

[B39] Wong MW Nordfors C Mossman D Pecenpetelovska G Avery-Kiejda KA Talseth-Palmer B Bowden NA Scott RJ BRIP1, PALB2, and RAD51C mutation analysis reveals their relative importance as genetic susceptibility factors for breast cancer Breast Cancer Res Treat 2011 127 853 859 10.1007/s10549-011-1443-0 21409391

[B40] Hellebrand H Sutter C Honisch E Gross E Wappenschmidt B Schem C Deissler H Ditsch N Gress V Kiechle M Bartram CR Schmutzler RK Niederacher D Arnold N Meindl A Germline mutations in the PALB2 gene are population specific and occur with low frequencies in familial breast cancer Hum Mutat 2011 32 E2176 E2188 10.1002/humu.21478 21618343

[B41] Medghalchi SM Frischmeyer PA Mendell JT Kelly AG Lawler AM Dietz HC Rent1, a trans-effector of nonsense-mediated mRNA decay, is essential for mammalian embryonic viability Hum Mol Genet 2001 10 99 105 10.1093/hmg/10.2.99 11152657

[B42] Mendell JT Medghalchi SM Lake RG Noensie EN Dietz HC Novel Upf2p orthologues suggest a functional link between translation initiation and nonsense surveillance complexes Mol Cell Biol 2000 20 8944 8957 10.1128/MCB.20.23.8944-8957.2000 11073994 PMC86549

[B43] Le Hir H Gatfield D Izaurralde E Moore MJ The exon-exon junction complex provides a binding platform for factors involved in mRNA export and nonsense-mediated mRNA decay Embo J 2001 20 4987 4997 10.1093/emboj/20.17.4987 11532962 PMC125616

[B44] Le Hir H Izaurralde E Maquat LE Moore MJ The spliceosome deposits multiple proteins 20-24 nucleotides upstream of mRNA exon-exon junctions Embo J 2000 19 6860 6869 10.1093/emboj/19.24.6860 11118221 PMC305905

[B45] Durand C Roeth R Dweep H Vlatkovic I Decker E Schneider KU Rappold G Alternative splicing and nonsense-mediated RNA decay contribute to the regulation of SHOX expression PLoS One 2011 6 e18115 10.1371/journal.pone.0018115 21448463 PMC3063249

[B46] Grandemange S Soler S Touitou I Expression of the familial Mediterranean fever gene is regulated by nonsense-mediated decay dagger Hum Mol Genet 2009 18 4746 4755 10.1093/hmg/ddp437 19755381

[B47] Danckwardt S Neu-Yilik G Thermann R Frede U Hentze MW Kulozik AE Abnormally spliced beta-globin mRNAs: a single point mutation generates transcripts sensitive and insensitive to nonsense-mediated mRNA decay Blood 2002 99 1811 1816 10.1182/blood.V99.5.1811 11861299

[B48] Amrani N Sachs MS Jacobson A Early nonsense: mRNA decay solves a translational problem Nat Rev Mol Cell Biol 2006 7 415 425 16723977 10.1038/nrm1942

[B49] Amrani N Ganesan R Kervestin S Mangus DA Ghosh S Jacobson A A faux 3 '-UTR promotes aberrant termination and triggers nonsense-mediated mRNA decay Nature 2004 432 112 118 10.1038/nature03060 15525991

[B50] Hogg JR Goff SP Upf1 senses 3 ' UTR length to potentiate mRNA decay Cell 2010 143 379 389 10.1016/j.cell.2010.10.005 21029861 PMC2981159

[B51] Eberle AB Stalder L Mathys H Orozco RZ Muhlemann O Posttranscriptional gene regulation by spatial rearrangement of the 3 ' untranslated region PLoS Biol 2008 6 849 859 10.1371/journal.pbio.0060092 PMC2689704 18447580

[B52] Ivanov PV Gehring NH Kunz JB Hentze MW Kulozik AE Interactions between UPF1, eRFs, PABP and the exon junction complex suggest an integrated model for mammalian NMD pathways Embo J 2008 27 736 747 10.1038/emboj.2008.17 18256688 PMC2265754

[B53] Oliver AW Swift S Lord CJ Ashworth A Pearl LH Structural basis for recruitment of BRCA2 by PALB2 Embo Rep 2009 10 990 996 10.1038/embor.2009.126 19609323 PMC2750052

[B54] Sy SMH Huen MSY Zhu YY Chen JJ PALB2 regulates recombinational repair through chromatin association and oligomerization J Biol Chem 2009 284 18302 18310 10.1074/jbc.M109.016717 19423707 PMC2709360

[B55] Sy SMH Huen MSY Chen JJ MRG15 Is a novel PALB2-interacting factor involved in homologous recombination J Biol Chem 2009 284 21127 21131 10.1074/jbc.C109.023937 19553677 PMC2755835

[B56] Dray E Etchin J Wiese C Saro D Williams GJ Hammel M Yu X Galkin VE Liu D Tsai MS Sy SM Schild D Egelman E Chen J Sung P Enhancement of RAD51 recombinase activity by the tumor suppressor PALB2 Nat Struct Mol Biol 2010 17 1255 1259 10.1038/nsmb.1916 20871616 PMC2950913

[B57] Hayakawa T Zhang F Hayakawa N Ohtani Y Shinmyozu K Nakayama J Andreassen PR MRG15 binds directly to PALB2 and stimulates homology-directed repair of chromosomal breaks J Cell Sci 2010 123 1124 1130 10.1242/jcs.060178 20332121 PMC2844321

[B58] Jones S Hruban RH Kamiyama M Borges M Zhang X Parsons DW Lin JC Palmisano E Brune K Jaffee EM Iacobuzio-Donahue CA Maitra A Parmigiani G Kern SE Velculescu VE Kinzler KW Vogelstein B Eshleman JR Goggins M Klein AP Exomic sequencing identifies PALB2 as a pancreatic cancer susceptibility gene Science 2009 324 217 10.1126/science.1171202 19264984 PMC2684332

[B59] Slater EP Langer P Niemczyk E Strauch K Butler J Habbe N Neoptolemos JP Greenhalf W Bartsch DK PALB2 mutations in European familial pancreatic cancer families Clin Genet 2010 78 490 494 10.1111/j.1399-0004.2010.01425.x 20412113

[B60] Tischkowitz MD Sabbaghian N Hamel N Borgida A Rosner C Taherian N Srivastava A Holter S Rothenmund H Ghadirian P Foulkes WD Gallinger S Analysis of the gene coding for the BRCA2-interacting protein PALB2 in familial and sporadic pancreatic cancer Gastroenterology 2009 137 1183 1186 10.1053/j.gastro.2009.06.055 19635604 PMC3914669

[B61] Dansonka-Mieszkowska A Kluska A Moes J Dabrowska M Nowakowska D Niwinska A Derlatka P Cendrowski K Kupryjanczyk J A novel germline PALB2 deletion in Polish breast and ovarian cancer patients BMC Med Genet 2010 11 20 20122277 10.1186/1471-2350-11-20 PMC2829009

[B62] Prokofyeva D Bogdanova N Bermisheva M Zinnatullina G Hillemanns P Khusnutdinova E Dork T Rare occurrence of PALB2 mutations in ovarian cancer patients from the Volga-Ural region Clin Genet 2012 82 100 101 10.1111/j.1399-0004.2011.01824.x 22310028

[B63] García MJ Fernández V Osorio A Barroso A Llort G Lázaro C Blanco I Caldés T de la Hoya M Ramón Y Cajal T Alonso C Tejada MI San Román C Robles-Díaz L Urioste M Benítez J Analysis of FANCB and FANCN/PALB2 Fanconi anemia genes in BRCA1/2-negative Spanish breast cancer families Breast Cancer Res Treat 2009 113 545 551 10.1007/s10549-008-9945-0 18302019

[B64] Tischkowitz M Xia B PALB2/FANCN: recombining cancer and Fanconi anemia Cancer Res 2010 70 7353 7359 10.1158/0008-5472.CAN-10-1012 20858716 PMC2948578

[B65] Heikkinen T Karkkainen H Aaltonen K Milne RL Heikkila P Aittomaki K Blomqvist C Nevanlinna H The breast cancer susceptibility mutation PALB2 1592delT is associated with an aggressive tumor phenotype Clin Cancer Res 2009 15 3214 3222 10.1158/1078-0432.CCR-08-3128 19383810

[B66] Ghadirian P Robidoux A Zhang P Royer R Akbari M Zhang S Fafard E Costa M Martin G Potvin C Patocskai E Larouche N Younan R Nassif E Giroux S Narod SA Rousseau F Foulkes WD The contribution of founder mutations to early-onset breast cancer in French-Canadian women Clin Genet 2009 76 421 426 10.1111/j.1399-0004.2009.01277.x 19863560

[B67] Hofstatter EW Domchek SM Miron A Garber J Wang M Componeschi K Boghossian L Miron PL Nathanson KL Tung N PALB2 mutations in familial breast and pancreatic cancer Fam Cancer 2011 10 225 231 10.1007/s10689-011-9426-1 21365267 PMC3836668

[B68] Papi L Putignano AL Congregati C Piaceri I Zanna I Sera F Morrone D Genuardi M Palli D A PALB2 germline mutation associated with hereditary breast cancer in Italy Fam Cancer 2010 9 181 185 10.1007/s10689-009-9295-z 19763884

[B69] Cao AY Huang J Hu Z Li WF Ma ZL Tang LL Zhang B Su FX Zhou J Di GH Shen KW Wu J Lu JS Luo JM Yuan WT Shen ZZ Huang W Shao ZM The prevalence of PALB2 germline mutations in BRCA1/BRCA2 negative Chinese women with early onset breast cancer or affected relatives Breast Cancer Res Treat 2009 114 457 462 10.1007/s10549-008-0036-z 18446436

[B70] Bogdanova N Sokolenko AP Iyevleva AG Abysheva SN Blaut M Bremer M Christiansen H Rave-Frank M Dork T Imyanitov EN PALB2 mutations in German and Russian patients with bilateral breast cancer Breast Cancer Res Treat 2011 126 545 550 10.1007/s10549-010-1290-4 21165770 PMC3291835

[B71] Erkko H Xia B Nikkilä J Schleutker J Syrjäkoski K Mannermaa A Kallioniemi A Pylkäs K Karppinen SM Rapakko K Miron A Sheng Q Li G Mattila H Bell DW Haber DA Grip M Reiman M Jukkola-Vuorinen A Mustonen A Kere J Aaltonen LA Kosma VM Kataja V Soini Y Drapkin RI Livingston DM Winqvist R A recurrent mutation in PALB2 in Finnish cancer families Nature 2007 446 316 319 10.1038/nature05609 17287723

